# Combining multi-OMICs information to identify key-regulator genes for pleiotropic effect on fertility and production traits in beef cattle

**DOI:** 10.1371/journal.pone.0205295

**Published:** 2018-10-18

**Authors:** Pablo Augusto de Souza Fonseca, Samir Id-Lahoucine, Antonio Reverter, Juan F. Medrano, Marina S. Fortes, Joaquim Casellas, Filippo Miglior, Luiz Brito, Maria Raquel S. Carvalho, Flávio S. Schenkel, Loan T. Nguyen, Laercio R. Porto-Neto, Milton G. Thomas, Angela Cánovas

**Affiliations:** 1 University of Guelph, Department of Animal Biosciences, Centre for Genetic Improvement of Livestock, Guelph, Ontario, Canada; 2 Universidade Federal de Minas Gerais, Departamento de Biologia Geral, Belo Horizonte, Minas Gerais, Brazil; 3 CSIRO Agriculture and Food, Queensland Bioscience Precinct, Brisbane, Queensland, Australia; 4 University of California-Davis, Department of Animal Science, Davis, California, United States of America; 5 The University of Queensland, School of Chemistry and Molecular Biosciences, Brisbane, Queensland, Australia; 6 Universitat Autònoma de Barcelona, Departament de Ciència Animal i dels Aliments, Barcelona, Bellaterra, Barcelona, Spain; 7 Canadian Dairy Network, Guelph, Ontario, Canada; 8 Colorado State University, Department of Animal Science, Fort-Colins, Colorado, United States of America; University of Florida, UNITED STATES

## Abstract

The identification of biological processes related to the regulation of complex traits is a difficult task. Commonly, complex traits are regulated through a multitude of genes contributing each to a small part of the total genetic variance. Additionally, some *loci* can simultaneously regulate several complex traits, a phenomenon defined as pleiotropy. The lack of understanding on the biological processes responsible for the regulation of these traits results in the decrease of selection efficiency and the selection of undesirable hitchhiking effects. The identification of pleiotropic key-regulator genes can assist in developing important tools for investigating biological processes underlying complex traits. A multi-breed and multi-OMICs approach was applied to study the pleiotropic effects of key-regulator genes using three independent beef cattle populations evaluated for fertility traits. A pleiotropic map for 32 traits related to growth, feed efficiency, carcass and meat quality, and reproduction was used to identify genes shared among the different populations and breeds in pleiotropic regions. Furthermore, data-mining analyses were performed using the Cattle QTL database (CattleQTLdb) to identify the QTL category annotated in the regions around the genes shared among breeds. This approach allowed the identification of a main gene network (composed of 38 genes) shared among breeds. This gene network was significantly associated with thyroid activity, among other biological processes, and displayed a high regulatory potential. In addition, it was possible to identify genes with pleiotropic effects related to crucial biological processes that regulate economically relevant traits associated with fertility, production and health, such as *MYC*, *PPARG*, *GSK3B*, *TG* and *IYD* genes. These genes will be further investigated to better understand the biological processes involved in the expression of complex traits and assist in the identification of functional variants associated with undesirable phenotypes, such as decreased fertility, poor feed efficiency and negative energetic balance.

## Introduction

The regulation of complex traits involves multiple *loci*, that contribute to a small proportion of the phenotypic expression, and environmental effects [1, 2]. In addition, *loci* that regulate complex traits are known to be involved in the regulation of several phenotypes. This describes the primary genetic effect that leads to genetic correlation, known as pleiotropy. Currently, the interaction and regulation pattern of these *loci* are poorly understood, especially in livestock species. However, the use of pleiotropic markers for genomic selection may result in improvement in efficiency of selection. On the other hand, some pleiotropic markers can lead to the indirect selection of undesirable traits. The exclusion of these markers from genetic selection programs may result in the improvement of a specific trait without affecting other traits or multiple traits simultaneously.

Indirect selection for undesirable traits has been well described in livestock breeding programs. For example, variants associated with high productive performance can also reduce fertility or affect mortality rates in both beef and dairy production systems [3–6]. The identification of *loci* with pleiotropic effects and the dissection of the biological processes in which these loci are involved can provide useful information to improve the accuracy of genomic breeding values by providing insights on the best statistical model to be used. Thus, more accurate breeding values translates in better breeding decisions, which will lead to reduced frequency of undesirable genotypes and phenotypes in the population under selection. Additionally, the use of causal and functional variants in selection models may result in a higher prediction accuracy and improved prediction persistence over generations [7–9].

The investigation of genomic *loci* with pleiotropic effects involved in the regulation of economically important traits is a key step to apply the breeding strategies previously mentioned. The detection of such genomic regions enables the identification of potential causal variants mapped in key regulator genes. Multivariate trait analysis and subsequent selection indexes may then be enhanced by the identification of these variants, leading to higher genetic improvement [10–12].

The use of functional genomics in a systems biology approach is a powerful tool for the integration and analysis of biological networks using high-throughput OMICs technologies [13, 14]. The integration of results from different breeds and OMICs approaches helps unravel the relationship between candidate genes and several phenotypes to further identify genetic variants associated with changes in the expression of those genes.

Fertility and production traits are good examples of phenotypes with distinct genetic architecture, different potential to respond to selection, and with antagonistic genetic relationship. The majority of fertility traits have unfavorable genetic correlations with production traits, which may be explained by the unidirectional selection, “hitchhiking” effect or pleiotropic effects [15–17]. The biological process responsible for sexual maturity, known as puberty, involves complex pathways that are regulated by several genes involved with the development of a wide variety of phenotypes [18]. Crucial biological structures for puberty development, e.g., hypothalamus, pituitary gland and thyroid, are involved in the regulation of synthesis and secretion of several hormones directly related to production traits [19–21]. Therefore, a deep investigation of the biological processes related to genes involved in puberty may result in the identification of key regulator genes for both fertility and production traits. Within this context, the aim of this study was to integrate multi-OMICs data into studies that investigated pubertal status and fertility traits. This integration was performed using a systems biology approach to identify candidate genes with pleiotropic effects on economically relevant traits in beef cattle.

## Material and methods

### Ethics statement

This study was carried out analysing data from previous studies, which have been approved by respective ethics in research committees [13, 20, 22]. Therefore, additional animal welfare and use committee approval was not required.

### Data collection

Candidate genes identified in three independent populations (i.e., breeds) of beef cattle were used. For the first population (Brangus cattle, n = 64), genes differentially expressed (DE) between pre- and post- puberty in eight tissues (hypothalamus, pituitary gland, liver, *longissimus dorsi* muscle, adipose tissue, uterine horn, endometrium, and ovary) were evaluated [13]. The candidate genes for the second population (Tropical Composite cattle, n = 866; Brahman = 843) were identified by a genome-wide association analysis (GWAS) for age at puberty, postpartum anestrous interval, and occurrence of the first postpartum ovulation before weaning in the first rebreeding period [22]. For the third population (Brahman cattle, n = 24), DE genes were identified in pre- and post- puberty stages in the pituitary gland and ovary [20].

### Identification of genes near pleiotropic markers

A total of 9,194 genes identified in the three breeds (2,120 in Brangus, 3,714 in Tropical Composite, and 5,269 in Brahman) were mapped against a list of genomic markers (n = 729,068) with pleiotropic effect (P < 0.05 after false discovery rate (FDR) correction–n = 21,908 markers) reported by Bolormaa *et al*. (2014) [10], which analyzed 32 traits including growth, feed intake, carcass and meat quality, and reproduction. Using the list of candidate genes obtained by integrating and combining data from OMICs technologies described by Cánovas *et al*. (2014) [13], Hawken *et al*. (2012) [22] and Nguyen *et al*. (2017) [20], genes up to 1 Mb (downstream and upstream) from a pleiotropic marker were selected. This interval was selected based on the average recombination block and the linkage disequilibrium (LD) pattern across the cattle genome [23, 24].

### Genes shared among breeds and QTL mapping

The genes identified within the pleiotropic regions were compared and those shared across the three independent populations were selected to be mapped against QTL regions using resources from the Cattle QTL database (CattleQTLdb, www.animalgenome.org/cgi-bin/QTLdb/BT/index). Using an interval of 2 Mb (1 Mb downstream and 1 Mb upstream) from each one of the shared genes, all the QTLs mapped in this interval were annotated. Those genes mapped in regions where at least five QTL categories were annotated (from six possible categories: exterior appearance, health, reproduction, production, meat and carcass and milk traits) were ranked as the genes with the highest pleiotropic potential.

### Functional analyses

**Fig 1:**
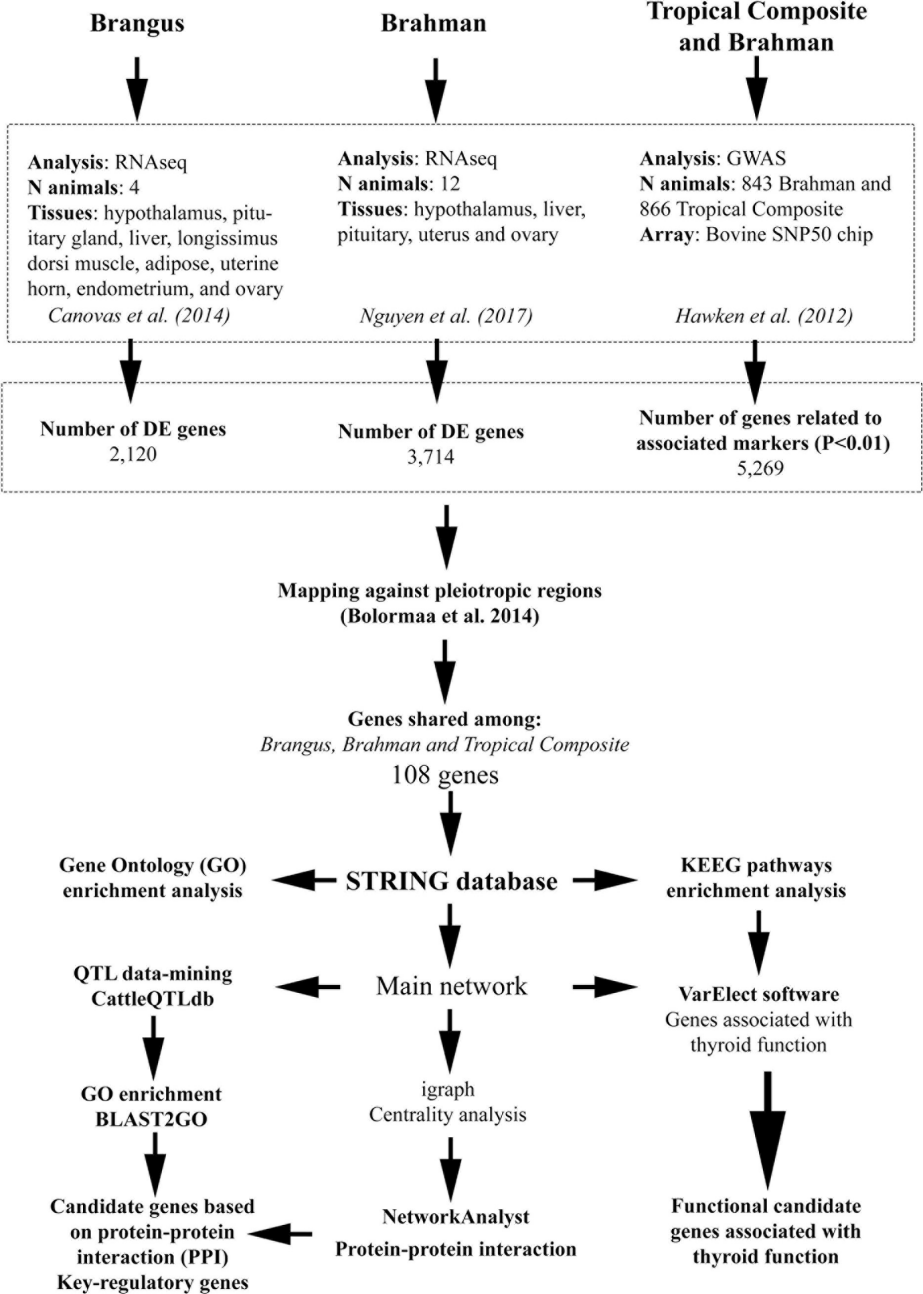
Flowchart presenting the methodological pipeline performed to identify the potential key-regulatory genes for pleiotropic effect on fertility and production traits in beef cattle.

First, the relationships among pleiotropic genes were estimated using literature text-mining, co-expression, gene fusion, protein homology, gene-neighborhood and gene co-occurrence using STRING database [25]. This estimation used human database, as the available information is more complete for humans than bovine. Additionally, using STRING database, an enrichment analysis was also performed to identify associations between the pleiotropic genes with biological processes (BP) and metabolic pathways using the Kyoto Encyclopedia of Genes and Genomes (KEGG). From the gene network created using STRING database, a centrality analysis was performed in order to identify the pleiotropic genes with the highest number of connections in the network. The *igraph* package [26] was used to calculate the centrality coefficient, defined as the number of connections of each node in a network. The VarElect software [27] was used to match the genes present in this list to the enriched KEGG pathways. In addition, for all the genes present in the main network, BLAST2GO [28] was used to obtain a better description of the BP that these pleiotropic genes are related to. Finally, the NetworkAnalyst tool [29] was used to confirm the genes with the highest regulatory potential based on the interaction with other proteins. The identification of the interactions among the genes was performed using IMEx, based on a literature-curated comprehensive data from InnateDB [30]. All the analyses described previously are shown in Fig 1.

## Results and discussion

**Fig 2:**
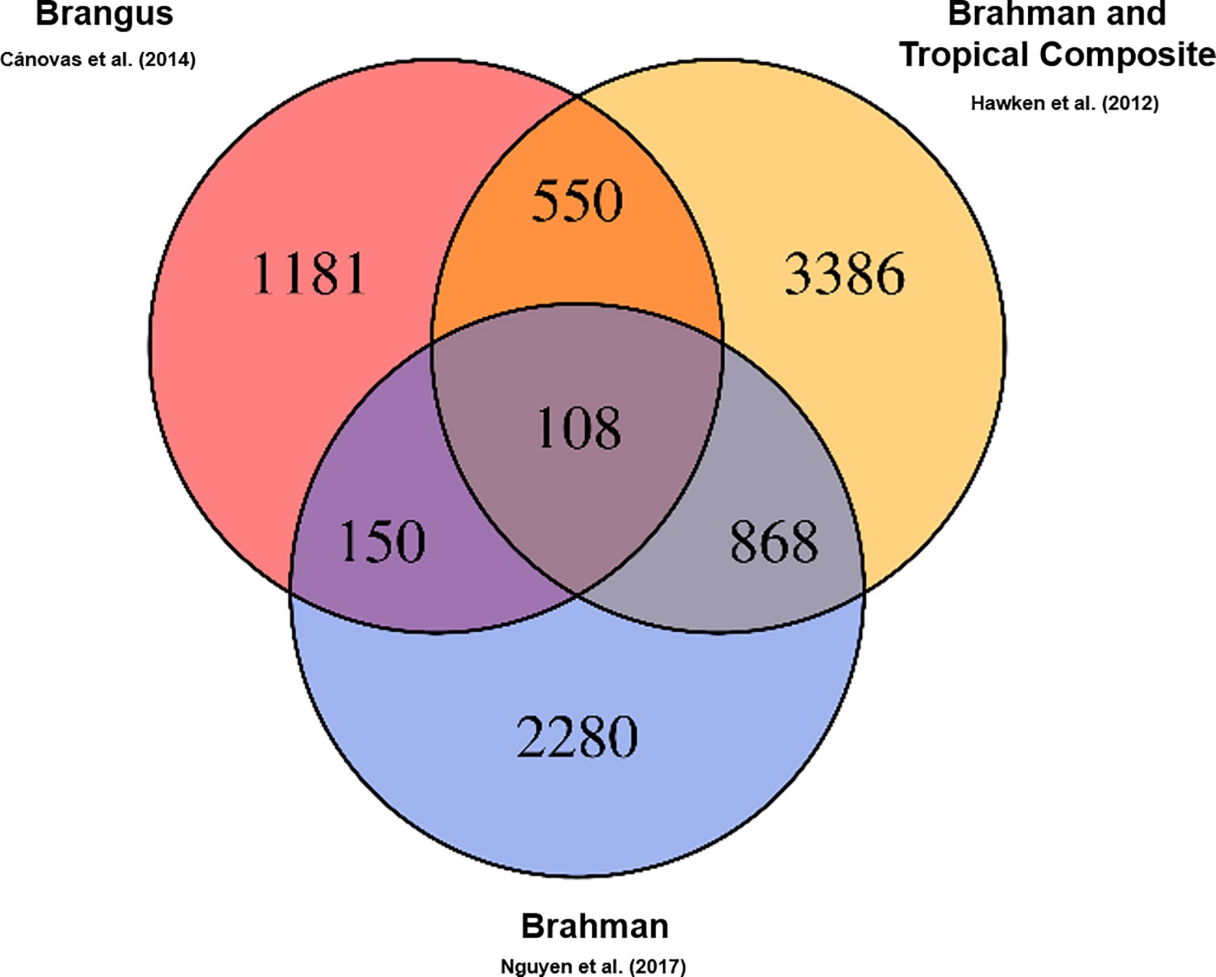
Venn diagram displaying the comparison of genes among the different independent populations analysed. In yellow, the genes identified in Brahman and Tropical Composite breeds (Hawken *et al*., 2012) [22]. In blue, the genes identified in Brahman breed (Nguyen *et al*., 2017) [20]. In red, the genes identified in Brangus breed (Cánovas *et al*., 2014) [13].

**Fig 3:**
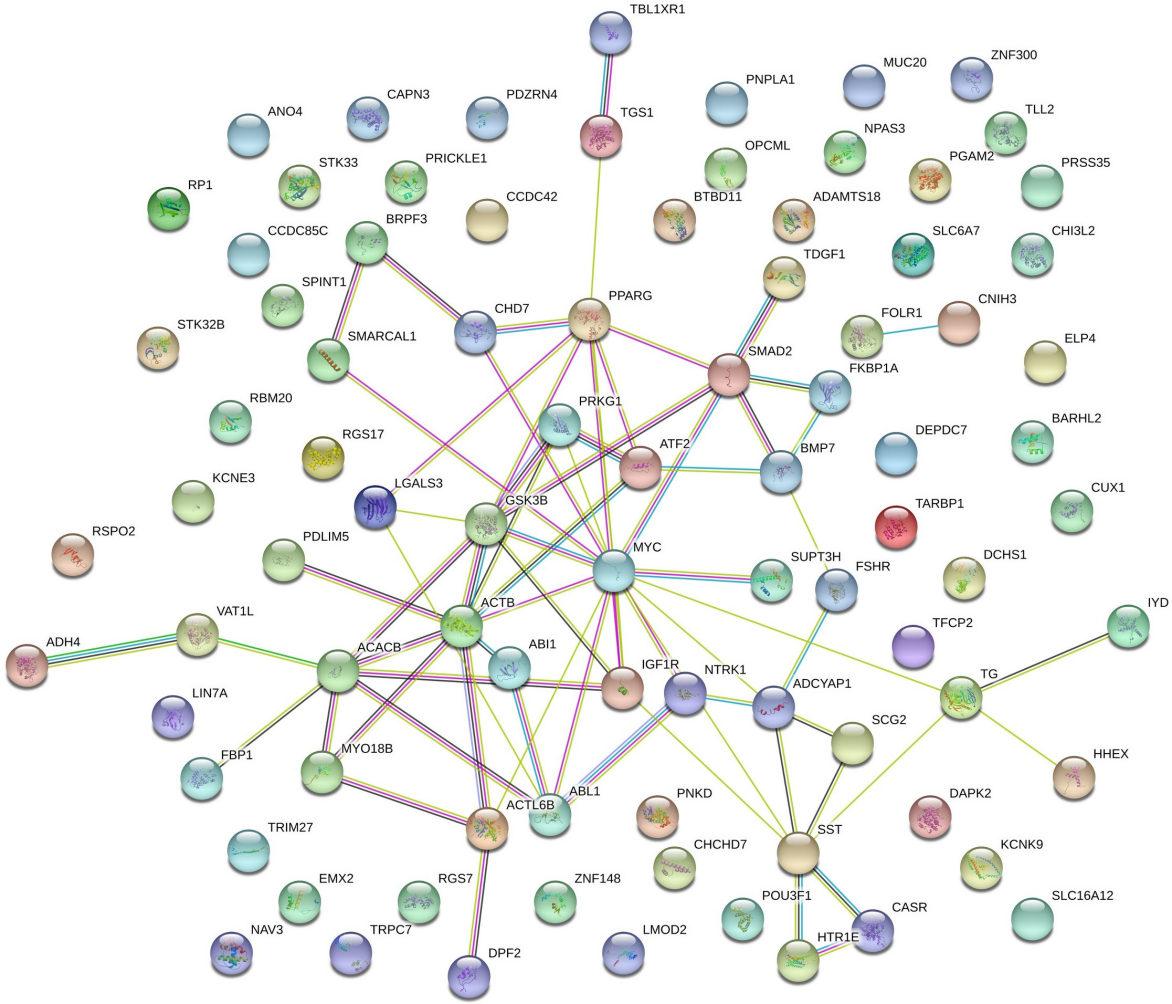
Gene network displaying the connections between the markers shared among the three independent populations and mapped in pleiotropic regions. The nodes represent individual genes. The coloured lines linking the nodes represents the interactions between the genes. The interactions between the genes can be divided in three types: 1) Known interaction: from curated databases (light blue) and experimentally determined (purple); 2) Predicted interactions: gene neighbourhood (green), gene fusions (red) and gene co-occurrence (dark blue); 3) Others: text mining (yellow), co-expression (black) and protein homology (violet). The interactions were based on human data, since the human database is more curate and complete than the bovine database.

**Fig 4:**
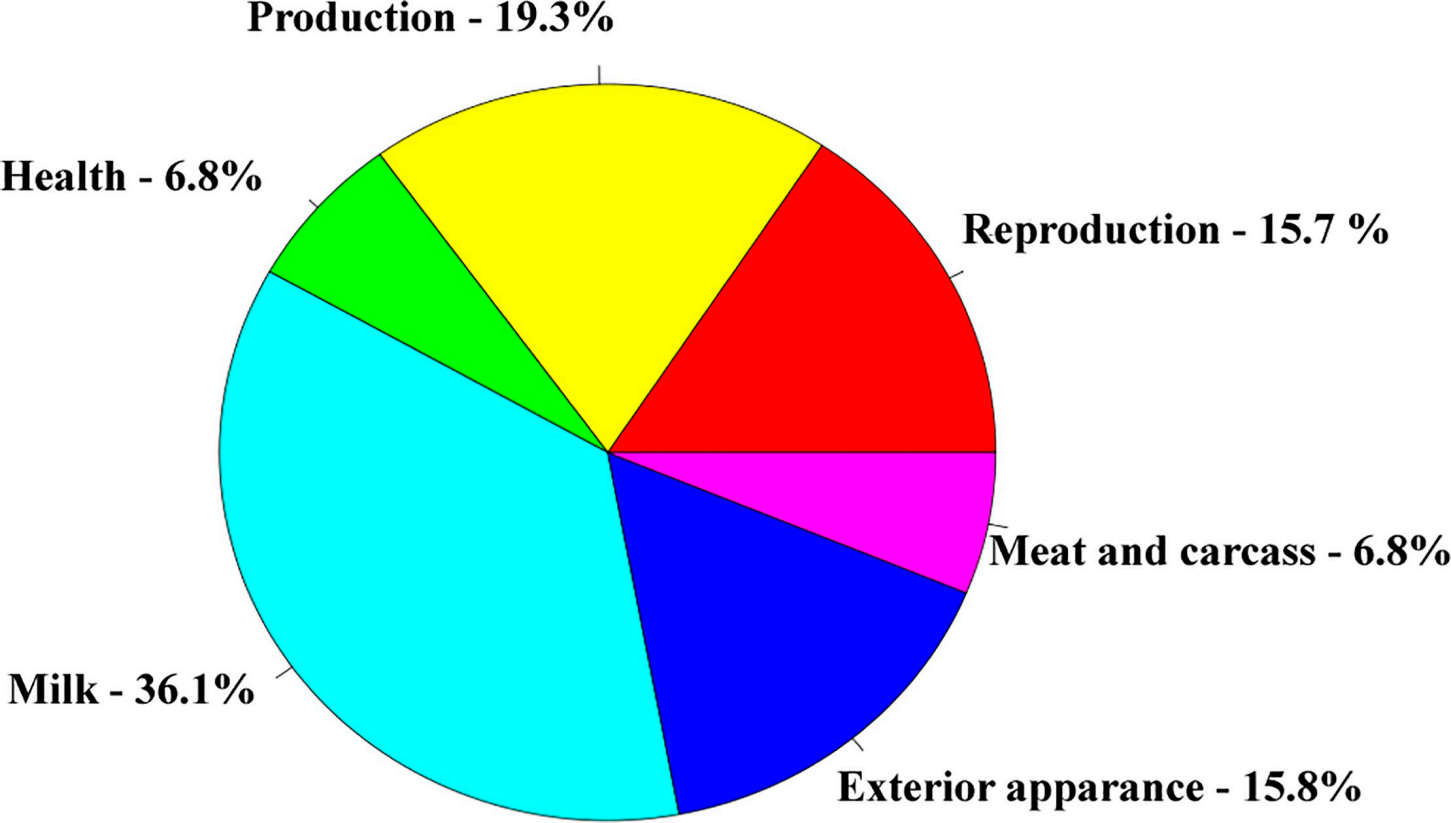
Proportion of each QTL category (reproduction, production, milk, meat and carcass quality, exterior appearance, and health) mapped in a 2 Mb interval (1Mb upstream and 1 Mb downstream) from each gene present in the main network (38 genes).

**Fig 5:**
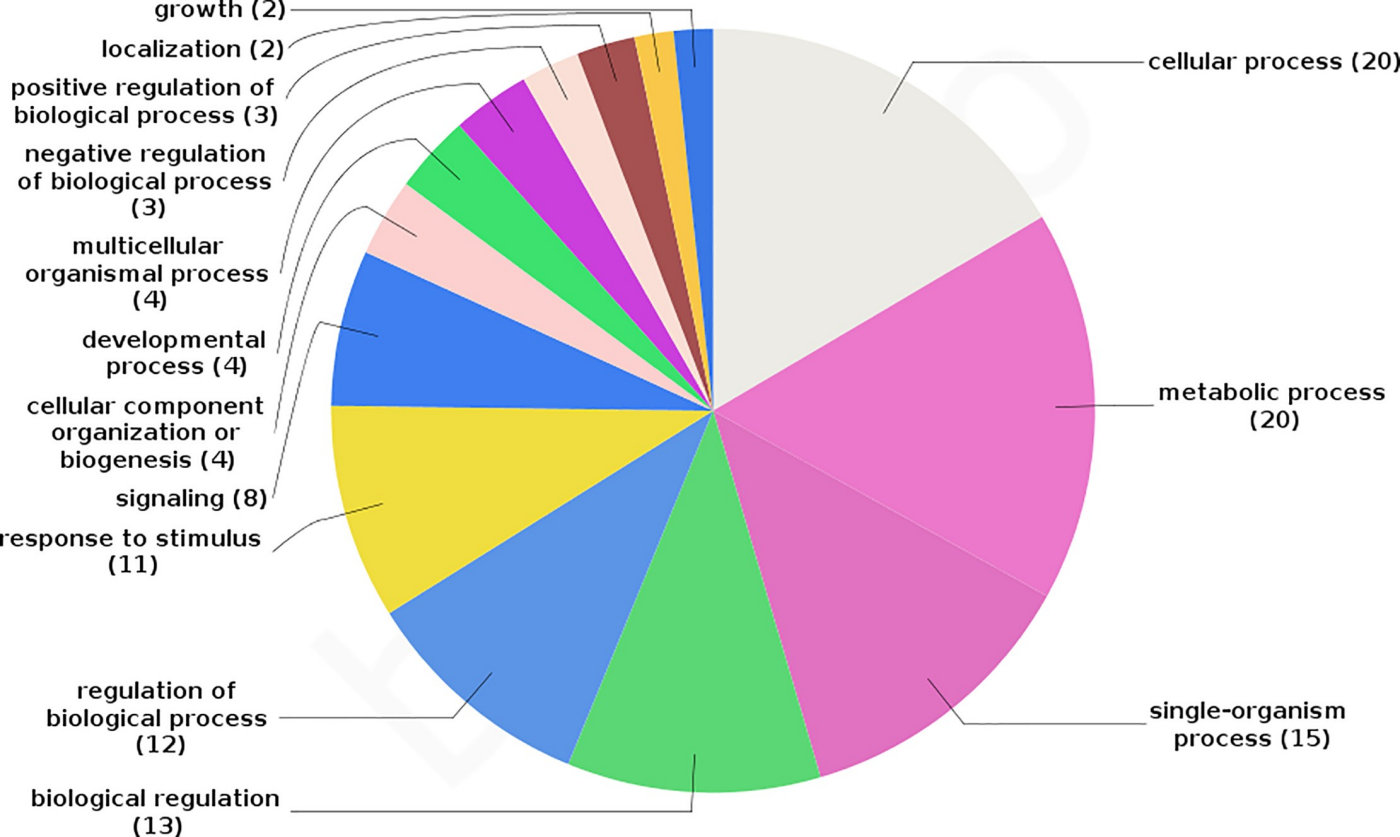
Biological processes (BP) significantly enriched in the main network identified by STRING database. The number inside the parenthesis indicated the number of genes associated with each BP.

**Fig 6:**
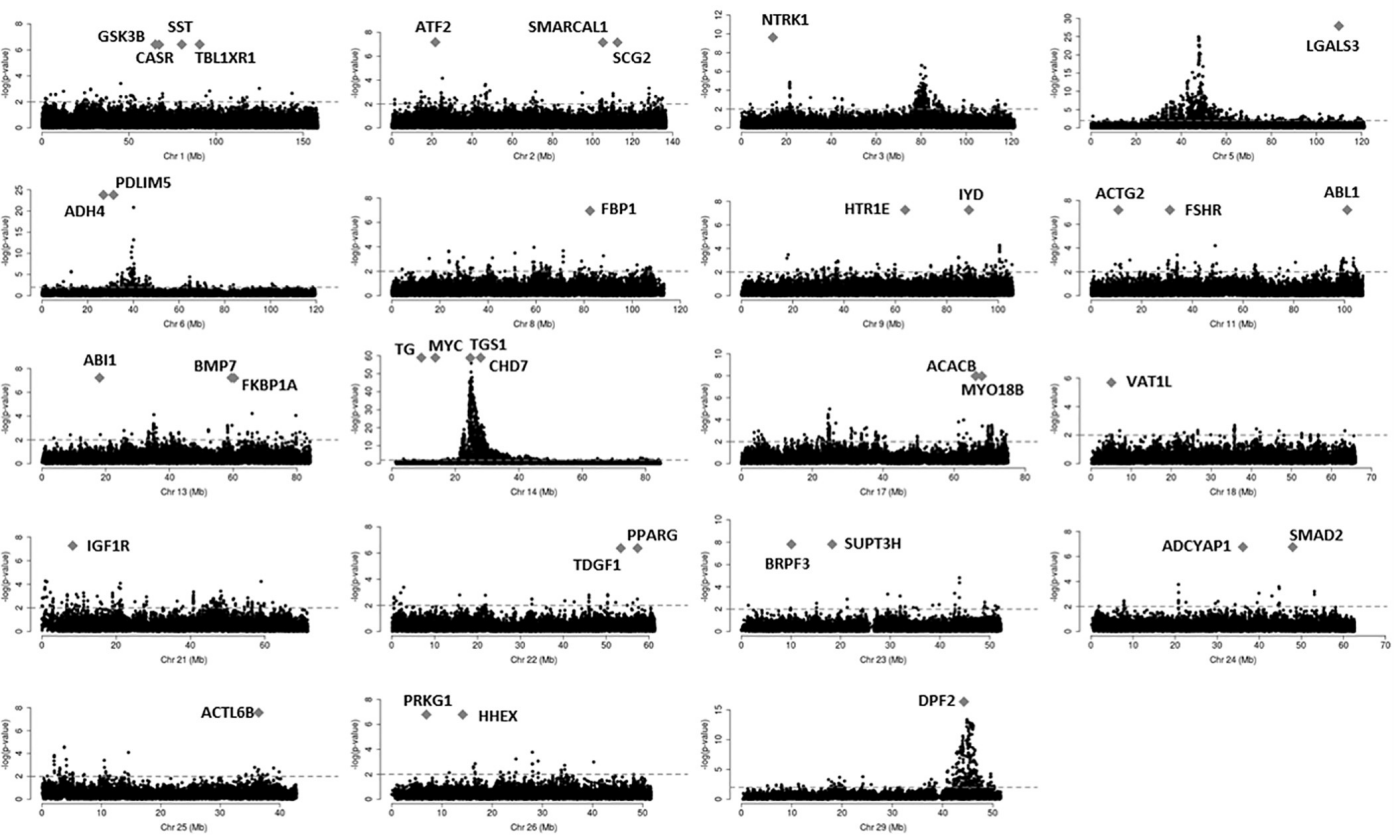
Chromosome specific plots displaying pleiotropic effect around the genes shared among all breeds. The x-axis corresponds to the genomic position in each chromosome and the y-axis to the -log(p-value). The -log(p-value) showed in the y-axis corresponds to the p-values adjusted to multiple-testing (FDR<0.01) obtained by Bolormaa et al. (2014) [10] for the pleiotropic analysis. The grey diamond corresponds to the start coordinate of each gene. The horizontal dashed lines indicate the nominal threshold of -log(p-value)>2. All the genes mapped in an interval of 1 Mb of a marker with significant signal for pleiotropic effect were considered as a genes in pleiotropic regions.

A total of 108 genes (S1 File) mapped in pleiotropic regions were shared among all three independent populations of the three beef cattle breeds considered (Fig 2). Among them, 89 genes were mapped in regions with five or six QTL categories annotated (S2 File). It is important to highlight that the approach to evaluate the post-puberty stage was different between Cánovas et al. (2014) [13] and Nguyen et al. (2017) [20]. The puberty period was defined as the second consecutive day of a circulating progesterone values >1 ng/mL by Cánovas et al. (2014) [13]. On the other hand, Nguyen et al. (2017) [20] defined the puberty period when the observation of the first corpus luteum occurred. These differences, together with the different genetic backgrounds and environmental effects between these two populations, can result in different expression profiles, even when two similar groups of phenotypes are measured (pre- and post-puberty). However, the same biological processes are expected to underlying the puberty development in both populations. Therefore, it is expected that the key-regulators of these processes maintained a similar DE profile between pre- and post-puberty phenotypes in both populations. Additionally, the consistency of expression among tissues and populations can help to identify these key-regulatory genes. The results obtained from BP and KEGG pathways enrichment analyses indicated that these genes are involved in development and maturation of the body (Table 1). From those 89 genes, 38 were grouped in the main network identified by STRING database (Fig 3 and S1 File). Fig 4 shows the percentage of each type of QTL (exterior appearance, health, reproduction, production, meat and carcass quality and milk traits) present in a 2 Mb interval (1 Mb downstream and 1 Mb upstream) around these 38 genes. The expression pattern across the tissues analyzed by Cánovas *et al*. (2014) [13] and Nguyen *et al*. (2017) [20] reinforce the functional relevance of these genes due to the DE across specific tissues (Table 2). The detailed mapping of the BP associated with these 38 genes suggested a strong relationship between these genes and the regulation of biological processes involved with cellular metabolism, cellular growth and development (Fig 5 and S3 File). Fig 6 shows the genomic context of the regions where these 38 genes were mapped as a function of the pleiotropic markers harboring these genes. The results from evaluation of the gene list composed of 38 genes suggested that the identification of key-regulatory genes was an important step to elucidate the relationship among these genes and the regulation of BP, as well as their association with economically important traits.

**Table 1:** Top 10 enriched biological processes (BP) and enriched KEGG pathways identified by STRING database using the 89 genes shared among the reported results from Cánovas et al. (2014) [13], Hawken et al. (2012) [22] and Nguyen et al. (2017) [20] and mapped in regions with 5 or more categories of QTL.

Top 10 enriched GO (Biological Function)			
ID	Description	Bonferroni P-value	Implicated genes
GO:0048513	Organ development	8.67E-05	ABI1, ABL1, ACTB, ACTL6B, ADAMTS18, ATF2, CAPN3, CCDC85C, CHD7, CUX1, DCHS1, EMX2, FKBP1A, FOLR1, FSHR, GSK3B, HHEX, IGF1R, LIN7A, MYC, NTRK1, POU3F1, PPARG, PRKG1, RBM20, RP1, RSPO2, SPINT1, TBL1XR1, TG, ZNF148
GO:0048731	System development	0.0002	ABI1, ACTB, ACTL6B, ADAMTS18, ADCYAP1, ATF2, BARHL2, BMP7, CAPN3, CCDC85C, CHD7, EMX2, FKBP1A, FOLR1, FSHR, GSK3B, HHEX, IGF1R, LGALS3, LIN7A, MYC, OPCML, PDLIM5, PPARG, PRICKLE1, PRKG1, RBM20, RP1, RSPO2, SCG2, SPINT1, TBL1XR1, TG, TRPC7, ZNF148
GO:0007275	Multicellular organismal development	0.0003	ABI1, ACTB, ACTL6B, ADAMTS18, ADCYAP1, ATF2, BARHL2, BMP7, CAPN3, CCDC85C, CHD7, EMX2, FKBP1A, FOLR1, FSHR, GSK3B, HHEX, IGF1R, LGALS3, LIN7A, MYC, OPCML, PDLIM5, PPARG, PRICKLE1, PRKG1, RBM20, RP1, RSPO2, SCG2, SMAD2, SPINT1, TBL1XR1, TG, TLL2, TRPC7, ZNF148
GO:0048856	Anatomical structure development	0.0003	ABI1, ACTB, ACTL6B, ADAMTS18, ADCYAP1, ATF2, BARHL2, BMP7, CAPN3, CASR, CCDC85C, CHD7, EMX2, FKBP1A, FOLR1, FSHR, GSK3B, HHEX, IGF1R, LIN7A, LMOD2, MYC, OPCML, PDLIM5, PPARG, PRICKLE1, PRKG1, RBM20, RP1, RSPO2, SCG2, SMAD2, SPINT1, TBL1XR1, TG, TRPC7, ZNF148
GO:0044707	Single-multicellular organism process	0.0004	ABI1, ACTB, ACTL6B, ADAMTS18, ADCYAP1, ATF2, BARHL2, BMP7, BRPF3, CAPN3, CASR, CCDC85C, CHD7, EMX2, FKBP1A, FOLR1, FSHR, GSK3B, HHEX, IGF1R, LGALS3, LIN7A, LMOD2, MYC, NPAS3, OPCML, PDLIM5, PGAM2, PNKD, PPARG, PRICKLE1, RBM20, RP1, RSPO2, SCG2, SMAD2, SPINT1, SST, TBL1XR1, TG, TLL2, TRPC7, ZNF148
GO:0009888	Tissue development	0.0007	ABI1, ATF2, CHD7, CUX1, DCHS1, FKBP1A, FOLR1, GSK3B, HHEX, IGF1R, LGALS3, MYC, NTRK1, POU3F1, PPARG, PRICKLE1, RP1, RSPO2, SPINT1, TBL1XR1, TDGF1
GO:0032502	Developmental process	0.001	ABI1, ACTB, ACTL6B, ADAMTS18, ADCYAP1, ATF2, BARHL2, BMP7, CASR, CCDC85C, CHD7, EMX2, FKBP1A, FOLR1, FSHR, GSK3B, HHEX, IGF1R, LIN7A, LMOD2, MYC, NPAS3, OPCML, PDLIM5, PPARG, PRICKLE1, PRKG1, RBM20, RP1, RSPO2, SCG2, SMAD2, SPINT1, TBL1XR1, TG, TLL2, TRPC7, ZNF148
GO:0060429	Epithelium development	0.001	ABI1, CHD7, CUX1, DCHS1, FOLR1, HHEX, IGF1R, LGALS3, MYC, NTRK1, POU3F1, PPARG, PRICKLE1, RSPO2, SMAD2, SPINT1
GO:0035239	Tube morphogenesis	0.003	BMP7, CHD7, DCHS1, FOLR1, HHEX, MYC, PRICKLE1, RSPO2, SMAD2, SPINT1
GO:0006468	Receptor activity	0.003	ABI1, ABL1, ATF2, DAPK2, GSK3B, IGF1R, MYC, NTRK1, PRKG1, SCG2, SMAD2, STK32B, STK33, TDGF1
**Enriched KEGG Pathways**			
KEGG: 5202	Transcriptional misregulation in cancer	0.00183	HHEX, IGF1R, MYC, NTRK1, PPARG, SPINT1, SUPT3H
KEGG: 5200	Pathways in cancer	0.00943	ABL1, DAPK2, GSK3B, IGF1R, MYC, NTRK1, PPARG, SMAD2
KEGG: 5216	Thyroid cancer	0.0193	MYC, NTRK1, PPARG
KEGG: 4390	Hippo signaling pathway	0.0286	ACTB, BMP7, GSK3B, MYC, SMAD2

**Table 2:** Inclusion criteria and/or expression pattern (differentially expressed), in Cánovas et al. (2014) [13] and Nguyen et al. (2017) [20], for the 38 genes mapped in the main network identified by STRING database.

Gene Symbol	Coordinate	Brangus (Cánovas et al., 2014) [13]											Tropical composite and Brahman (Nguyen et al., 2017) [20]	
		SNP	TS	TF	Hyp	Pit	Ov	Ut	End	Lv	Ad	ldm	Pit	Ov
GSK3B	1:65265851–65292071	X												X
CASR	1:67255165–67344655		X											X
SST	1:80250205–80251648		X			X	X	X		X		X		X
TBL1XR1	1:90428333–90609529			X										X
ATF2	2:21725894–21830562			X										X
SMARCAL1	2:105135339–105189135			X										X
SCG2	2:112549869–112555375		X											X
NTRK1	3:14019229–14037583					X		X						X
ADH4	6:26853175–26885436		X				X				X	X		X
PDLIM5	6:31333660–31568270	X												X
FBP1	8:82460863–82491694		X											X
HTR1E	9:63760625–63852410					X								X
IYD	9:88612923–88624698					X			X				X	
LGALS3	10:67843328–67861114		X											
FSHR	11:31110744–31305197		X											X
ABL1	11:101011169–101152856			X										X
ABI1	13:18094943–18182433	X												X
BMP7	13:59424987–59510393	X												X
FKBP1A	13:60276502–60303717	X											X	X
TG	14:9253697–9263933					X		X					X	
MYC	14:13769242–13775688			X										X
TGS1	14:24747192–24772996	X												X
CHD7	14:28043739–28172246	X												X
ACACB	17:66101179–66217542		X										X	
MYO18B	17:67768278–68002792		X											X
VAT1L	18:5045212–5211278	X											X	
IGF1R	21:7967701–8268340	X												X
TDGF1	22:53432382–53437166		X		X	X		X	X	X				X
PPARG	22:57367072–57432321		X	X										X
BRPF3	23:10096561–10132302			X										X
SUPT3H	23:18223167–18623659			X										X
ADCYAP1	24:36114443–36121104					X						X		X
SMAD2	24:47963393–48022086			X										X
ACTL6B	25:36459674–36469794		X											X
ACTB	25:39343633–39347047		X											
PRKG1	26:6901760–8343635	X											X	
HHEX	26:14120258–14126069			X										X
DPF2	29:44205003–44217694	X		X										X

SNP: Genes expressed in at least one tissue among the two physiological states (pre- and post-puberty) and mapped near the markers associated with fertility traits by GWAS; TF: transcriptional factor; TS: genes identified with high probability to show a binding site for TF differentially expressed; Hyp: hypothalamus; Pit: pituitary; Ov: ovary; Ut: uterus; End: endometrium; Lv: liver; Ad: adipose tissue; ldm: *longissimus dorsi* muscle.

### Identification of key-regulatory genes through gene-network analysis

**Fig 7:**
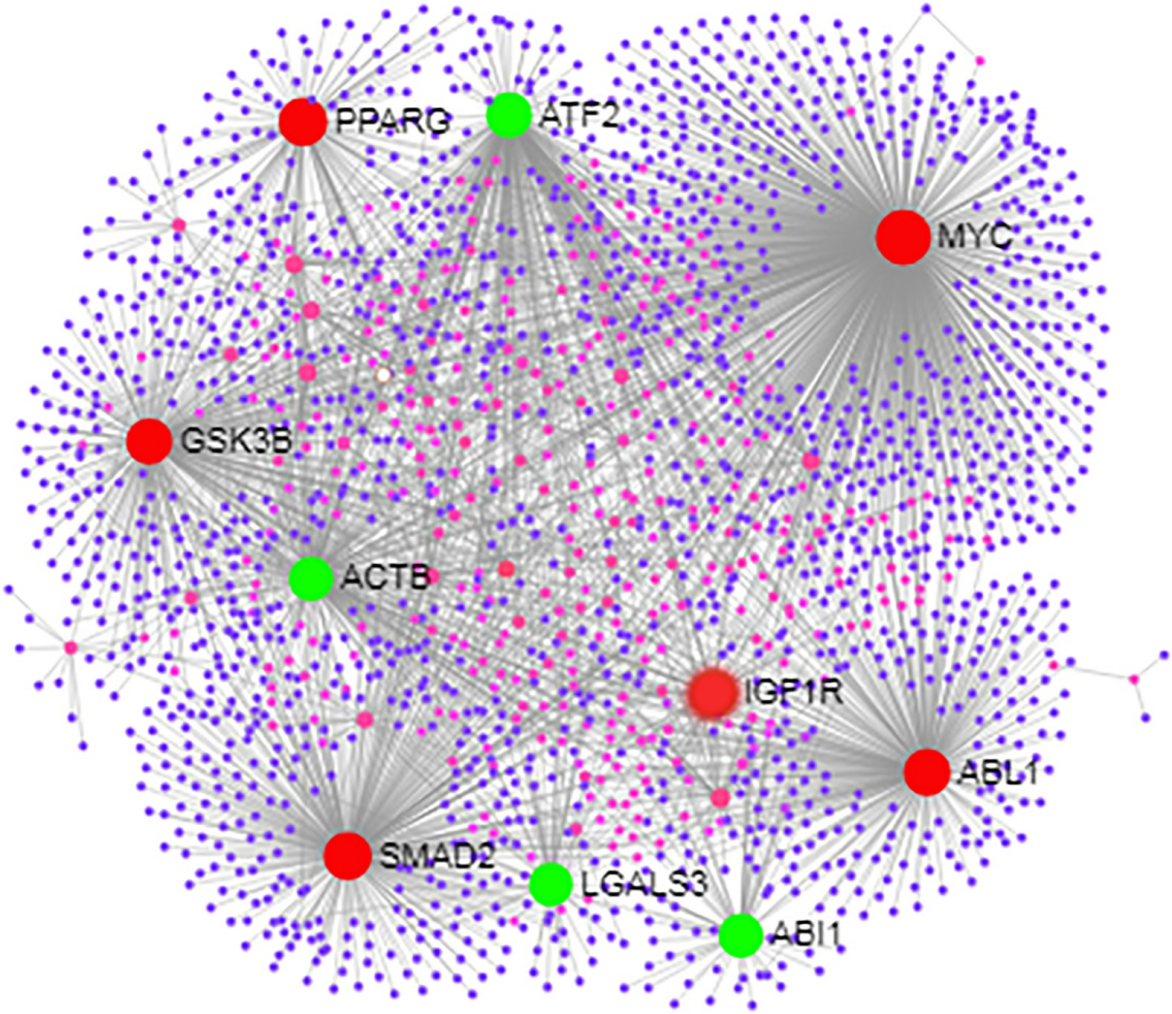
Interactome displaying the protein-protein interactions for the genes present in the main gene network identified by STRING database with other proteins across the genome. Larger nodes (highlighted in red and green) represent the genes with the highest number of connections. Genes in red are the genes associated with positive and negative regulation of cellular metabolic processes.

The centrality analysis for the main gene network, composed by 38 genes, identified the genes with the largest number of connections in the network. Table 3 shows the number of connections for the top 10 genes with the largest number of interactions. In order to confirm and reinforce the identification of genes with the highest regulatory potential, the interaction pattern of these 38 genes with other proteins was evaluated using IMEx. From this analysis, it was observed that there was an overlap between the top 10 genes with more interactions in the STRING database network and the top 10 genes from the IMEx interactions. In the IMEx analyses, it was also possible to identify, from the top 10 genes, six genes directly related to positive and negative regulation of cellular metabolic processes (red circles in Fig 7). Interestingly, these 6 genes were also present in the list of top connected genes in the main network identified by STRING database. These results confirmed the regulatory potential of these genes and highlight the biological processes in which these genes were involved. These genes were: MYC proto-oncogene (*MYC)* Peroxisome Proliferator Activated Receptor Gamma (*PPARG*), Glycogen Synthase Kinase 3 Beta (*GSK3B*), SMAD Family Member 2 (*Smad2*), ABL Proto-Oncogene 1, Non-Receptor Tyrosine Kinase (*ABL1*) and Insulin-like growth factor 1 receptor (*IGF1R*).

**Table 3:** Top 10 genes based on the number of interactions identified in the STRING database and NetworkAnalyst analyses. In bold are shown the genes present in both top 10 lists.

Top 10 genes for numbers of interactions with other genes			
STRING database		NetworkAnalyst	
Gene symbol	Number of interaction	Gene symbol	Number of interaction
**MYC**	14	**MYC**	714
**ACTB**	9	**SMAD2**	313
**GSK3B**	8	**ABL1**	276
**PPARG**	8	**GSK3B**	243
SST	7	ATF2	241
ACACB	7	**ACTB**	198
**ABL1**	5	**PPARG**	124
**SMAD2**	5	ABI1	64
ADCYAP1	5	**IGF1R**	58
**IGF1R**	5	LGALS3	56

Three of these genes are involved in cell proliferation: *MYC*, *SMAD2* and *ABL1*. *MYC* was the gene with the highest number of interaction with other genes in both network analyses. This gene codes for a transcription factor responsible for regulating transcription of several genes. Consequently, *MYC* plays a multifunctional action involved with the control of crucial biological processes, such as cell cycle control and cellular transformation [31]. The expression of *MYC* was decreased in the muscle tissue of orchidectomized testosterone-treated male mice, indicating that this gene might be involved with the promotion of muscle mass by maintaining myoblasts in the proliferative state; and with the differentiation and growth of muscle tissue in a process mediated by androgen receptors [32]. Additionally, *MYC* was identified as playing a crucial role in the regulation of gene networks during development of the lactation cycle and meat and carcass traits in cattle [33–35].

*SMAD2* is a member of the TGF-beta-SMAD signaling pathway and it is involved with the regulation of several processes associated with female reproduction and embryonic development in cattle [36]. In male rats, *SMAD2* is DE between non-sexually mature and sexually mature rats, indicating a relationship with puberty progression [37]. The expression of myostatin, a protein involved with muscle proliferation, is directly regulated by *SMAD2* activity [38–40]. Therefore, *SMAD2* is a crucial regulator of processes involved with fertility and production traits.

*ABL1* is a proto-oncogene associated with the regulation of several biological processes related to cellular division and differentiation. *ABL1* was observed as DE in mouse testis after heat shock, indicating a regulatory activity in this tissue [41]. Additionally, homozygous disruptions of *ABL1* are associated with neonatal lethality in mice [42]. To our best knowledge, the association between *ABL1* and production traits in cattle has been poorly investigated. However, a significant peak associated with angularity in Brown Swiss cattle was identified close to *ABL1* region. These results suggested a potential association between *ABL1* with fertility and production and conformation traits, reinforcing the necessity to improve our understanding of these relationships.

Two of the other six genes identified in this analysis are involved in energy conservation metabolism: *PPARG*, *GSK3B*. PPARG belongs to a subfamily of nuclear receptors involved in several crucial biological processes, such as adipogenesis and immune cell activation [43]. Alterations in the expression pattern of *PPARG*, usually decrease in expression, were already associated with puberty progression and fertility traits in several species, including humans and cattle [44–46]. Additionally, *PPARG* has also been associated with meat quality and, more specifically, intramuscular fat percentage, and milk synthesis in cattle, which are economically important traits in beef and dairy cattle production [47–49], respectively.

Muscle glycogen is absolutely fundamental as energy reservoir. The amount of liver and muscle glycogen available determines the use of fat and, as a last resource, amino acids, for providing energy. *GSK3B* is a serine-threonine kinase member of a subfamily of glycogen synthase kinase and its corresponding function is known to be associated with energy metabolism, body pattern formation and neuronal cell development [50]. Additionally, *GSK3B* has been associated with age at puberty, sperm motility and decrease in spermatogenesis. Therefore, its function is directly related to fertility traits [51–53]. Interestingly, *GSK3B* has also been associated with several economically relevant traits such as skeletal muscle hypertrophy, intramuscular fat, meat quality, milk synthesis and mammary gland proliferation [54–57].

The last gene in this list, *IGF1R*, is the receptor of the Insulin-like growth factor 1 (*IGF1*), which also binds *IGF2* and insulin with lower affinity. *IGF1* and *IGF2* have remarkable functions in the steroidogenic activity and regulation of body growth and maturation. In female cattle, *IGF1* and *IGF2* are associated with the regulation of the steroidogenic activity in the glanulosa cells, as well as, the regulation of the mitosis (*IGF1* and *IGF2*) and apoptosis during the follicular development (*IGF1*) [58, 59]. The bovine testis is an important source of *IGF1* production [60]. Both circulating and locally produced (testis) *IGF1* may play a crucial role in the testicular size and testosterone secretion [61, 62]. The *IGF1* pathway influence gonadotrophin‐releasing hormone (GnRH) neurons during the puberty and it is directly associated with the puberty progression and the development of reproductive traits [52, 63, 64]. Additionally, *IGF1* was associated with the regulation of several production traits, for example, feed intake, feed conversion, body weight, milk protein yield, milk fat yield, milk fat concentration and somatic cell score [65–67]. Variants mapped on *IGF1R* may result in a change of affinity between *IGF1* and its receptor, resulting in a different response to the circulating levels of this hormone. The crucial roles of *IGF1* in the regulation of body development, in addition to the results obtained in the present study, highlights the potential of *IGF1R* to act like a key-regulator of pleiotropic effects associated with fertility and production traits.

All the six genes described above are directly related to fertility and economically relevant traits. Additionally, these genes were identified as potential key-regulatory genes due to the number of interactions with other genes and its biological functions. Consequently, these genes are important candidate genes for pleiotropic effect on multiple production and fertility traits.

Genes that do not appeared in both analyses described in Table 3 were not included in the discussion. However, some of these genes are fundamental to the metabolic processes discussed here. For example, somatostatin (SST), also known as growth hormone inhibiting hormone (GHIH), affects energy conservation metabolism by inhibiting insulin and glucagon secretions. In addition, somatostatin produced in the hypothalamus is transported to anterior pituitary, where it inhibits the release of growth hormone (GH), TSH and prolactin [68]. This gene also encodes neuronostatin, which is also implicated in the releasing of pituitary hormones [69]. In addition, somatostatin is involved in the MAPK, cAMP, PKA- pathways, among many other ones [70]. As a consequence, somatostatin has also a role in energy conservation metabolism and cell proliferation, the two main biological processes detected in the current study.

*ACACB* and *ADCYAP1* are involved in the energy conservation metabolism. *ACACB* encodes acetil-CoA carboxilase, the enzyme converting acetil-CoA in malonyl-CoA, fundamental for fatty acids biosynthesis (KEGG ID: 04931; ID: 00061). *ADCYAP1* encodes adelylate-clycase interacting protein 1, which through the cAMP and PKA pathways is involved in the insulin upregulation and in the regulation of insulin levels in insulin secretory granules, respectively, among other functions (KEGG ID: 04911).

*ABI1* and *ATF2* are cell proliferation regulators. *ABI1* encodes ABL interacting protein 1, which facilitates the ABL cell proliferation signal. *ATF2* encodes activating transcription factor 2, also known as *CREB2*. *CREB2* regulates cell cycle proteins, pro-apoptotic proteins, cell adhesion molecules, and membrane and cytoplasm signaling proteins. In additions, CREB2 is the final step in many pathways, including cAMP, PKA, estradiol 17-beta (KEGG ID: 04915) and glucagon (KEGG ID: 04922). Therefore, *CREB2* is also involved in the energy conservation metabolism.

*LGALS3* encodes galectin 3, a carbohydrate binding protein with affinity for beta-galactosides. As a consequence, galectin 3 is involved in cell proliferation, adhesion, differentiation, angiogenesis and apoptosis [71]. It has been shown that galectin 3 is expressed by trophoblast cells in response to 17b-estradiol, progesterone, and human chorionic gonadotropin (hCG). Among many other effects, galectin 3 induces apoptosis in endometrial cells, which would allow embryo implantation [72].

### Thyroid function and genes with pleiotropic effect

Among the genes present in the main network identified by STRING database, there are two crucial genes for the synthesis of thyroid hormones. These genes are thyroglobulin (*TG*) and iodotyrosine deiodinase (*IYD*). *TG* is metabolized through the addition of iodine molecules to produce mono- and di-iodotyrosine and the thyroid hormones triiodothyronine (T3) and thyroxine (T4). Consequently, *TG* is one of the main storage molecules of iodine in the body [73]. *IYD* is responsible to conserve iodide, recycling iodine, during synthetization of T3 and T4 hormones, from mono- and diiodotyrosine. Due to the low availability of iodine in the nature, IYD dysfunctions reduce the amount of available iodine for T3 and T4 synthesis [74–76]. In target cells, IYD converts T4 to T3, the active thyroid hormone, and converts T3 to di-iodotyrosine, inactivating T3. These processes also provide iodine to peripheral tissues. The thyroid hormones are related to the control of several crucial biological processes involved with the regulation of basal metabolism. In cattle, genes related to thyroid hormone regulation have been identified as DE in studies evaluating feed efficiency, lactation stage, fat deposition and early embryonic development [77–83]. Additionally, some studies suggest a pleiotropic effect for TG polymorphisms in production traits [84, 85]. Additionally, changes in the levels of thyroid hormones during pregnancy, mainly in the initial development of the embryos, are associated with adverse pregnancy outcomes and embryonic losses [86]. Moreover, alterations in thyroid activity may result in male and female infertility [19, 87].

An interesting link between the thyroid hormones and the selection for productive traits in cattle is that *TG* (BTA14:9,253,697–9,263,933) is mapped in the same core selective sweep (CSS) region of *DGAT1* (BTA14: 1,795,425–1,804,838) [88]. The QTL related to *DGAT1* is considered to have a major effect on production traits and has been associated with several phenotypes in both dairy and beef cattle breeds [89–91]. Due to this major effect, molecular markers associated with the *DGAT1* effect are intensively exploited in genetic improvement programs. Generally, the use of molecular markers in selection programs does not consider the relationship among the aimed marker and the surrounding mapped markers. However, the intensive selection may increase the extent and the overall LD in a region [92]. This phenomenon may result in an indirect selection (hitchhiking effect) of markers mapped in different genes and with unpredictable effects. It is important to note that, in the same CSS, the highest signal for pleiotropic effect was observed by Bolormaa *et al*. (2014) [10], as shown in Fig 6. In the same position, three additional genes shared among the three independent populations mapped to regions with 5 or more previously reported QTLs and in the main network identified by STRING database, i.e. *MYC*, *TGS1* and *CHD7*.

The thyroid cancer pathway (KEEG ID: 05216) was one of the enriched pathways identified by STRING database (Table 1). In this pathway, thyroid hormones and precursors are not involved, but both MYC and PPARG are implicated, reinforcing the association between this main-network and the regulation of thyroid activity. In addition to the presence of *TG* and *IYD* in the main network identified by STRING database (Fig 3), these results suggest that this network is enriched by genes related to thyroid function. The VarElect software was used to confirm this hypothesis through an association processes between the genes that composed the main network and the keywords “thyroid” and “thyroid hormones”. Through data-mining using information available on GeneCards and MalaCards, it was possible to identify that from the 38 genes (S4 File) present in the main network, 31 are directly related to those keywords. Therefore, indicating that this gene network was enriched by genes related to thyroid function.

As previously described, thyroid function is related to the regulation of several biological processes associated with economically important traits. Additionally, due to this association with several processes and traits, the genes involved in thyroid activity are excellent functional candidate genes for pleiotropic effects. Further functional analyses will be performed in order to elucidate the relationship between the different traits affected by pleiotropic effects, as well as, to identify candidate variants associated with the function of these candidate genes.

It is important to highlight that during the analyses performed here, some differences were observed in the gene expression profile among populations, even when similar groups were compared (Pre- and post-puberty). A very common phenomenon observed in biological analyses that can help to address this issue is the Simpson’s paradox. The Simpson’s paradox is observed when results from aggregated data contradict those from separate analyses. There are several reports in the literature discussing the impact of Simpson’s paradox in different fields, such as network analysis and gene expression [93, 94, 95]. The biological bases for the Simpson’s paradox in biological analyses are still poorly understood. However, some points can be raised to help in the discussion of this phenomenon. For example, in the data analysed in the present manuscript, as addressed in the previous commentary, the differences in the genetic background, environmental effects and evaluation of the puberty, and number of tissues evaluated between populations can help to explain these differences. It is important to highlight that these populations (Brangus, Brahman and Tropical composition) shared the Brahman genetic component. However, these proportions are different in each population. For example, in the Brangus population the animals have 3/8 of Brahman component and 5/8 of Angus component. All these differences can be taken together in order to discussion the possible causes of the phenomenon observed here. Additionally, it is important to highlight that the genes shown in Table 2 are a specific group of genes, which are the genes with the highest potential to perform pleiotropic effect. Additionally, the consistency of expression among tissues and populations can help to identify these key-regulatory genes. The present study aims exactly on those genes that even with all these possible confounding factors maintain the expression profile, which can be an additional evidence of crucial regulatory role.

## Conclusions

The present study described a multi-breed and multi-OMICs approach to identify key-regulatory candidate genes for pleiotropic effects in beef cattle using the results generated by previous studies. Our findings confirm the feasibility of using a systems biology approach to unravel candidate genes regulating complex traits. Genes identified in this study are mainly involved in two biological processes: energy conservation metabolism and cell proliferation, probably the most theoretically plausible processes to unify the phenotypes investigated in this study: exterior appearance, health, reproduction, production, meat and carcass, and milk traits. This study contributes to the understanding of the cause-consequence relationships between variants mapped on candidate pleiotropic genes affecting complex traits. Additionally, the results obtained here will be useful for better defining statistical models to improve the accuracy of genomic prediction of breeding values and avoid the simultaneous selection for unfavorable genetically correlated traits in beef cattle and other livestock species.

## Supporting information

S1 File Ensembl gene ID, official symbol and genomic coordinates for the 108 genes shared among all the populations (tab 1) and 38 genes present in the main network identified by STRING DB (tab 2). (XLSX)

S2 File QTL information for the 89 genes were mapped in regions with five or six QTL categories annotated. (TXT)

S3 File Gene ontology terms associated with the 38 genes in the main network identified by STRING DB. (TXT)

S4 File Varelect analysis output for the 38 genes in the main network identified by STRING DB. (XLSX)
